# Red Yeast Rice

**DOI:** 10.3390/foods6030019

**Published:** 2017-03-01

**Authors:** Thu Nguyen, Mitchell Karl, Antonello Santini

**Affiliations:** 1 Boca Raton Hospital Campus, Internal Medicine Residency Program, FAU/ Schmidt School of Medicine, Boca Raton, FL 33486, USA; karlm@health.fau.edu; 2 Department of Pharmacy, University of Napoli Federico II, Via D. Montesano 49, Napoli 80131, Italy; asantini@unina.it

**Keywords:** red yeast rice, lovastatin, nutraceutical, safety, health

## Abstract

Red yeast rice (RYR), produced by the fermentation of the *Monascus purpureus* mold, has been used for a long time in Asian cuisine and traditional medicine. It consists of multiple bioactive substances, including monacolins, which potentially can be used as a nutraceutical. Monacolin K, which is chemically identical to lovastatin, has been recognized as responsible for the cholesterol-reducing effect of this compound. While the European Food Safety Authority maintains that the use of monacolin K from RYR preparations of at least 10 mg can produce a normal blood cholesterol level, the United States Food and Drug Administration considers monacolin K, due to its similarity with lovastatin, an unapproved drug, and therefore marketing of products that label the monacolin content is prohibited. This mini-review summarizes the benefit of RYR in hyperlipidemia, maintains RYR use as a food, and addresses the importance of regulation regarding RYR and the need for clinical data and clear label information for consumers with reference to a toxin-free, non-augmented, standardized amount of monacolins.

## Red Yeast Rice as Nutraceutical

The term “nutraceutical” was coined in 1989 by Stephen DeFelice, founder and chairman of the Foundation for Innovation in Medicine, and it identifies a food or part of a food, which can be of vegetal or animal origin, that has a potential pharmaceutical activity [1]. The goal of assessing the possible role of a nutraceutical and its use in medicine is an important challenge for the future.

In general, any food, due to its content of active compounds, has the potential to go beyond its nutritional value as a source of macro- and micronutrients, and can also be used as a drug depending on the dose. Nonetheless, attention should be paid to potential risk factors related to the use of vegetal- or animal-origin foodstuffs as starting matrices to constitute a nutraceutical, e.g., the safety of the starting material, the presence of allergenic compounds, the absence of toxicity, the absence of exogenous and endogenous contaminants, the possible presence of toxic secondary metabolites and/or environmental pollutants, which could potentially cause a health threat [2,3].

Red yeast rice (RYR) is a nutraceutical made by fermenting white rice with the yeast *Monascus purpureus* and other related molds. RYR has been used as an herbal supplement and in the cuisine of East Asian countries including China, Japan, and Korea. It has been used for flavoring, coloring, and preservation of food and in traditional Chinese medicine for many years [4].

RYR consists of a multitude of compounds including polyketides, unsaturated fatty acids, phytosterols, pigments, and monacolins [5]. Monacolins inhibit HMG CoA (3-hydroxy-3-methyl-glutaryl-coenzyme A) reductase, the rate-limiting step in cholesterol synthesis. At least 13 monacolins have been isolated from RYR, of which monacolin K is chemically similar to lovastatin, a cholesterol-lowering drug [6].

Several clinical trials have been conducted to examine the efficacy and safety of RYR. A recent meta-analysis in 2015 examined 20 randomized trials consisting of 6663 patients and showed a reduction in low density lipoprotein (LDL) cholesterol when comparing RYR to placebo groups (−1.02 mmol/L (−1.20, −0.83)); there was no difference in LDL between RYR and statin therapy (0.03 mmol/L (−0.36, 0.41)), with an incidence of kidney injury and liver abnormalities of less than 5% in both the RYR and control groups [7]. RYR has been demonstrated to not only improve lipid metabolism, but it can also reduce blood pressure, and may possess anti-inflammatory, antidiabetic, anticancer, and osteogenic properties [5]. The largest randomized controlled trial examining RYR in secondary cardiovascular prevention consisted of 4870 Chinese patients and showed that RYR reduces nonfatal myocardial infarction, coronary disease mortality, coronary revasculization, and total mortality in patients with a history of myocardial infarction and moderate hypercholesterolemia [8].

Furthermore, a number of clinical trials have shown RYR to be effective in reducing cholesterol in those who are intolerant of statins because of statin-associated myalgias, gastrointestinal side effects, or elevated transaminase levels [4]. For those who are skeptical of drugs and are more interested in complementary and alternative medications, RYR has been used as a cholesterol-lowering option [9]. RYR in combination with other nutraceuticals including berberine, policosanol, astaxanthin, and coenzyme Q10 has been shown to be effective in reducing lipids and glucose. A recent meta-analysis of 14 randomized controlled trials showed that nutraceutical combinations containing RYR improve lipid and glucose levels [10]. Although statins have been shown to cause hyperglycemia, a 2014 meta-analysis of five trials consisting of 352 patients showed that RYR does not significantly increase glucose compared to placebo [8]. In addition, RYR in combination with antioxidants has been demonstrated to reduce high sensitivity C-reactive protein (hs-CRP) and endothelial dysfunction [11].

Adverse effects of RYR include gastrointestinal effects and may cause myopathy, hepatotoxicity, rhabdomyolysis, and anaphylaxis similar to the use of statins [9,12]. The mycotoxin citrinin, found in poorly produced RYR products, can pose a health risk as it may be mutagenic as found in animal models, genotoxic to human lymphocytes, and can cause kidney failure in animals, although acute toxicity is a rare event [13,14,15,16]. Furthermore, drug-herb interactions can potentially be harmful. Statins are metabolized by Cytochrome P450 (CYP) enzymes, and the administration of RYR with CYP enzyme inhibitors (e.g., ketoconazole, human immunodeficiency virus (HIV) protease inhibitors, erythromycin) can lead to worsening undesirable adverse effects, such as myopathy [5,17]. Nonetheless, the clinical studies that showed the effectiveness of RYR in dyslipidemia also demonstrated that it is a relatively safe product [7,18].

The European Food Safety Authority (EFSA) allows for health claims that RYR products can cause pharmacotherapeutic effects. The EFSA has established “a cause and effect relationship...between the consumption of monacolin K in red yeast rice preparations...and maintenance of normal blood LDL cholesterol concentrations”, given that the daily dietary intake level is at least 10 mg of monacolin K from RYR [19]. This value is considered acceptable even though recently it has been called into question due to the variable contents of different RYR products, thus representing a health risk in the absence of appropriate information for the customer [17].

The U.S. Food and Drug Administration (FDA) maintains a different perspective. Because of its functional similarity to lovastatin, monacolin K is considered an unapproved drug by the U.S. FDA, and as such all RYR products that contain a specific amount of monacolin K are prohibited. In fact, RYR supplements on the market are not standardized and contain variable amounts of monacolins, including monacolin K, citrinin, and other contaminants. Manufacturers do not admit the monacolin content in their RYR products on the packaging for fear it would prompt regulatory action from the FDA [9,20].

In order to reduce the variability of the monacolin contents of different RYR preparations and to minimize toxic compounds, such as citrinin, quality control should be implemented and enforced by entities responsible for food and supplement oversight. 

Due to its extensive use as a dietary supplement for many years before its medicinal purposes were discovered, RYR is a food and should be treated as such. Because of its wide potential health benefits, RYR should be produced as a standardized preparation for those who would potentially benefit from it. By not augmenting this product, it could be made available to consumers as a supplement and thus does not need to be rigorously regulated as a drug. Requiring a statement on the product label assuring a toxin-free, non-augmented, standardized amount of monacolins would be advantageous to consumers, allowing more predictable efficacy and better safety. In addition, a safety warning should also be included to caution those with myopathy, liver disease, or concomitant use of CYP inhibitors. Those who are taking this supplement should be advised to have regular follow-ups with a medical professional to monitor for potential side effects and interactions with drugs and other nutraceuticals.
